# Substance use and adolescent injuries: a multi-country analysis of the association and mediating effect of interpersonal violence among 122,945 in-school paediatric populations in 29 countries

**DOI:** 10.3389/fpubh.2023.1193711

**Published:** 2023-07-20

**Authors:** Benjamin Noble Adjei, Maxwell Afetor, Samuel Ansong-Aggrey, Reforce Okwei, Stephen Uwumbordo Nachibi, Lambongang Munkaila, Abdul Wahid Arimiyaw, Emmanuel Osei Bonsu, Collins Adu, Prince Peprah

**Affiliations:** ^1^Department of Epidemiology and Biostatistics, School of Public Health, Kwame Nkrumah University of Science and Technology, Kumasi, Ghana; ^2^Information, Monitoring and Evaluation Department, Ghana Health Service, Ho Polyclinic, Ho, Ghana; ^3^Department of Mathematics and Acturial Science, KNUST, Kumasi, Ghana; ^4^Department of Health Policy, Management and Economics, School of Public Health, Kwame Nkrumah University of Science and Technology, Kumasi, Ghana; ^5^Department of Geography, Miami University, Oxford, OH, United States; ^6^Department of Geography, Environment and Earth Sciences, University of Hull, Hull, United Kingdom; ^7^Department of Agribusiness and Applied Economics, North Dakota State University, Fargo, ND, United States; ^8^Department of Geography and Rural Development, Kwame Nkrumah University of Science and Technology, Kumasi, Ghana; ^9^College of Public Health, Medical and Veterinary Sciences, James Cook University, Townsville, QLD, Australia; ^10^Center for Social Research in Health, UNSW Sydney, Sydney, NSW, Australia; ^11^Social Policy Research Centre, UNSW, Sydney, NSW, Australia; ^12^Centre for Primary Health Care and Equity, UNSW, Sydney, NSW, Australia

**Keywords:** substance use, interpersonal violence, serious injuries, mediation, adolescents

## Abstract

**Background:**

Adolescent use of substances and injury experiences such as head injury have become increasingly prevalent. However, information regarding their association and the potential pathways linking them remains limited. This study examined the association between substance use and injuries, emphasizing the mediating role of interpersonal violence among adolescents.

**Methods:**

We employed a multi-country analysis of Global School-based Health Surveys of 122,945 in-school adolescents aged 11–18 from 29 countries. This study was a cross-sectional school-based, nationally representative study developed by the World Health Organization and the United States Centers for Disease Control and Prevention, other United Nations allies, and country-specific institutions. Random-effects meta-analysis was performed to estimate the overall prevalence of injury and substance use and the I-square ( $I^{2})$ statistic was used to investigate the between-country heterogeneity. Logistic regression models were fitted to examine the association between substance use and injuries. A path analysis was used to examine the potential mediation effect of interpersonal violence and employed decomposition of effects into total, direct, and indirect.

**Results:**

Prevalence of substance use and injuries were 33.6% (95%CI = 28.5, 38.6%) and 41.7% (95%CI = 37.3, 46.1%), respectively. Substance use (37.8% vs. 29.4%, *p* = 0.001) and injuries (47.3% vs. 36.4%, *p* = 0.001) were significantly higher among male adolescents than females, respectively. After adjustment, substance users had 40% higher odds of injuries. The path analysis showed a mediation effect of perpetration of and victimization by interpersonal violence on the association of substance use with injuries, with total positive effects of perpetration [*β =* 0.18; 95%CI = 0.16, 0.19; *p* = 0.001] and victimization on injuries [*β =* 0.22; 95%CI = 0.21, 0.24; *p* = 0.001]. In a further subgroup analysis, tobacco users were 3.98 times more likely to sustain a gunshot wound whiles marijuana users had 2.81 times higher odds of sustaining gunshot wounds. Cigarette smokers had 45% lower odds of sustaining cut/stab wounds. Alcohol users were 53% more likely to sustain concussion/head injury and two and half times more likely to sustain gunshot wound.

**Conclusion:**

A significant association exists between substance use and severe injuries among adolescents, mediated by interpersonal violence exposure. Our findings may have utility in informing substance use and interpersonal violence control policies and interventions to address adolescent injuries.

## Introduction

Substance use remains one of the adolescents’ main risk-taking lifestyle behaviours, potentially impacting their mental and physical health and well-being (1). Among adolescents, tobacco products and alcohol remain the most prevalent substances (2). These substances are primarily used before initiating illicit drugs such as marijuana (2). Specifically, regarding alcohol, more than a quarter of young people aged 15–19 years are current drinkers, which is estimated to affect 155 million adolescents worldwide (1, 3–5). Recent studies among adolescents found that 4.1% smoked cigarettes, 2% used forms of tobacco other than combustible cigarettes (eg. Tobacco products), and 38% used marijuana, respectively (5, 6).

Substance use increases public health issues, such as serious injuries (7). The prevalence of substance use-related injuries among adolescents is 42.2% in four southeast Asian countries (8) and 45.3% in sub-Saharan Africa [SSA] (9). Studies suggest that substance using adolescents are more likely to experience severe injuries (10, 11). For instance, a review of studies on injury risk associated with cannabis and cocaine use concluded that most intentional and general injuries were associated with the use of cannabis and cocaine (10). Research has found that persons who abused a wide range of licit and illicit substances were likelier to experience multiple injuries (12). Moreover, a significant association between traumatic brain injury (TBI) and substance use among young, incarcerated people have been reported (13).

Substance use and injuries are serious public health issues. However, there is a lack of knowledge regarding the potential pathways explaining the link between substance use and severe injuries among adolescents. Violence and aggression could link substance use to severe injuries (11). Inferring from preliminary evidence, interpersonal violence is mainly motivated by substance use (14), which could mediate substance use and serious injuries. Therefore, this study sought to explore the mediating effect of perpetration of and victimization by interpersonal violence on substance use and serious injuries. We hypothesized that perpetration of and victimization by interpersonal violence mediates the association between substance use and serious injuries.

## Methods

### Data source

This study used publicly available data from the Global School-based Student Health Survey (GSHS) among 29 countries and territories. The GSHS is a large and representative survey of students’ health behaviours and risk factors using a standardized two-stage probability sampling approach for participant selection. The survey was developed by the World Health Organization (WHO) and the US Centers for Disease Control and Prevention (CDC), and other United Nations (UN) allies and country-specific institutions (1, 15). The survey draws content from the CDC Youth Risk Behaviours Survey (YRBS), for which test–retest reliability has been established (16). Information, including the aims, methodology, and sampling procedure of the GSHS, is available at http://www.cdc.gov/gshs/. This study used the most recent data available in GSHS-participating countries and territories. Countries and territories with their most recent data released before 2015 were excluded from the analysis. Also, countries with recent datasets missing information on the variables of interest for the present study were excluded. By applying the inclusion and exclusion criteria, data from adolescents aged 11–18 years in 29 countries and territories were considered recent and eligible to be included in the analysis (see Table 1).

**Table 1:** Distribution of study sample across countries and territories.

Country	Year of survey	*N*	Unweighted %	Weighted %
Anguilla	2016	595	0.5	3.4
Argentina	2018	48,522	39.5	3.5
Benin	2016	1,775	1.4	3.5
Bhutan	2016	6,371	5.2	3.5
Cook Islands	2015	557	0.4	3.1
Curacao	2015	2,183	1.8	3.5
Dominican Republic	2016	1,057	0.9	3.4
Fiji	2016	2,848	2.3	3.4
French Polynesia	2015	2,529	2.1	3.5
Indonesia	2015	8,956	7.3	3.5
Jamaica	2017	1,226	1.0	3.5
Lao Republic	2015	3,028	2.5	3.5
Lebanon	2017	4,243	3.4	3.5
Liberia	2017	1,497	1.2	3.5
Mauritius	2017	2,207	1.8	3.4
Mozambique	2015	1,258	1.0	3.2
Nepal	2015	5,123	4.2	3.5
Philippines	2015	6,961	5.7	3.5
Samoa	2017	1,450	1.2	3.5
Seychelles	2015	1,755	1.4	3.5
Sri Lanka	2016	2,834	2.3	3.4
St Lucia	2018	1,421	1.2	3.5
Suriname	2016	1,561	1.3	3.4
Thailand	2015	3,952	3.2	3.5
Timor-Leste	2015	1,265	1.0	3.5
Trinidad and Tobago	2017	2,757	2.2	3.4
Tonga	2017	2,721	2.2	3.5
Vanuatu	2016	1,507	1.2	3.4
Wallis and Futuna	2015	786	0.6	3.5
Total		122,945	100.0	100.0

### Data collection procedure

Data collection was conducted during a regular class period with the help of trained enumerators from local social work organizations and other research institutions. The GSHS questionnaire was close-ended, structured and developed in English but translated into the local language in each country. The questionnaire included multiple-choice questions. After informed consent was obtained from the students, parents, and school officials, the questionnaires were administered. The trained enumerators explained the aim of the study and the instructions in detail to the students before the questionnaires were distributed. In addition, the survey ensured that students’ privacy was protected through anonymous and voluntary participation. The study complied with the Declaration of Helsinki, and each country’s Institutional review boards or ethics committees reviewed and approved all GSHS surveys.

### Assessment of substance use

The respondent’s substance use was assessed with single items for tobacco, marijuana, cigarette, and alcohol use. The items included “During the past 30 days, how many days did you smoke cigarettes?” (0 days, 1 or 2 days, 3 to 5 days, 6 to 9 days, 10 to 19 days, 20 to 29 days and all 30 days), “During the past 30 days, on how many days did you use any tobacco products other than cigarettes, such as country specific examples?” (0 days, 1 or 2 days, 3 to 5 days, 6 to 9 days, 10 to 19 days, 20 to 29 days and, all 30 days), “During the past 30 days, how many times have you used marijuana?” (1 = 0 times, 2 = 1 or 2 times, 3 = 3–9 times, 4 = 10–19 times and 5 = 20 or more times), and “During the past 30 days, how many days did you have at least one drink containing alcohol?” (0 days, 1 or 2 days, 3 to 5 days, 6 to 9 days, 10 to 19 days, 20 to 29 days and all 30 days). All variables were re-coded on a dichotomous scale (Yes or No, where 0 times/days were considered as No and at least 1 day/time was considered Yes) as in other existing GSHS studies (17–19). We created a composite variable, “substance use,” by assigning a score of “1” each for “Yes” responses to smoking cigarettes, other tobacco, marijuana, and alcohol. A “0” score was assigned if the response was “No”. The score for each participant was added with a possible total score of 0–4. Participants who scored ≥1 were considered substance users.

### Assessment of serious injuries

The primary outcome of this study was a self-reported serious injury. This variable was assessed in response to the question, “During the past 12 months, how many times were you seriously injured?” The GSHS questionnaire defined serious injury as an injury that makes the respondent miss at least one full day of usual activities such as school, sports, or a job or requires treatment by a doctor or nurse. Response options to the question were 1 = 0 times; 2 = 1 time; 3 = 2 or 3 times; 4 = 3 or 4 times; 4 = 4 or 5 times; 5 = 6 or 7 times; 6 = 8 0r 9 times; 7 = 10 or 11 times; 8 = 12 or more times. For analytic purposes, the options were further categorized into 1 = 0 [No] and 2 to 12 or more times = 1 [Yes] for this study. The students whose response option was “0 times” showed that they had not sustained any serious injury, while the remaining response options meant that they had sustained one or more injuries in the 12 months preceding the survey (9, 20, 21). We dichotomized the variable to simplify the analysis by analyzing group differences rather than individual differences (22).

### Assessment of interpersonal violence

Perpetration of and victimization by interpersonal violence were the mediating variables in this study. Perpetration of interpersonal violence was measured as “During the past 12 months, how many times were you in a physical fight?” and victimization by interpersonal violence was assessed as “During the past 12 months, how many times were you physically attacked?” The two variables had the same response options including 1 = 0 times; 2 = 1 time; 3 = 2 or 3 times; 4 = 4 or 5 times; 5 = 6 or 7 times; 6 = 8 or 9 times; 7 = 10 or 11 times; and 8 = 12 or more times. For simplification and analytic purposes, the responses were further categorized into “No” for those who responded “0 times,” with the remaining responses as “Yes” in both variables Previous studies have used perpetration of and victimization by interpersonal violence to measure interpersonal violence (20, 23, 24).

### Statistical analysis

The “svyset” command in Stata was used for the statistical analyses to adjust the complex sampling design employed by the GSHS survey using the weight, primary sampling unit (PSU), and stratum variables. Descriptive statistics were used to analyze and describe respondents’ characteristics stratified by substance use status. The chi-square test of independence was used to determine the association between substance use and background characteristics. Country-specific prevalence of substance use and injury was summarised using proportions and 95% confidence intervals. To calculate the overall prevalence of injury and substance use, we performed random-effects meta-analysis in stata. We investigated the between-country heterogeneity using the I-square ( $I^{2})$ statistic and the high heterogeneity ( $I^{2}$ >95%) was used to justify the random-effect modelling. Bivariate and multivariable logistic regression models were used to assess the influence of substance use on injuries, adjusting for other risk factors of injury. Odds ratios and 95% confidence intervals were reported. Variables with a value of *p* of 0.05 or less were considered statistically significant in the multivariable analysis. A path analysis using the structural equation model examined the potential mediation effect of perpetration of and victimization by interpersonal violence on the association between substance use and injury. Direct and indirect effects were tested, and the decomposition of total, direct, and indirect effects were specified (25). A sub-group analysis was also conducted to determine the associations between substance types and forms of injuries. This analysis employed a multinomial logistic regression analysis, with injury types as the outcome variable and substance types (tobacco, cigarette, marijuana, and alcohol) as the explanatory variables. Broken bone/dislocated joint was considered the base outcome, while concussion/head injury, gunshot wound, bad burn, and poison were the other outcomes used for the multinomial regression analysis. The data were analyzed from multiple countries jointly, and a country variable was included in the regression models to account for the joint analysis derived from multiple surveys.

## Results

### Background characteristics of the respondents by substance use

Table 2 shows the background characteristics of the respondents by substance use. In general, the majority of respondents were below the age of 15. Females constituted a higher proportion at 52.0%. The majority of the respondents (78.6%) reported having three or more close friends. A significant percentage, 68.0%, engaged in physical activity, while 79.5% received support from peers. Approximately 45.4% experienced feelings of loneliness. Around one-third of respondents reported being bullied, 9.2% had a suicidal plan, 8.1% had attempted suicide, and roughly 9.6% had contemplated suicide. Aside from hunger, all other background characteristics (age, sex, number of close friends, physical activity, peer support, loneliness, worry, bullying, suicidal plan, suicidal attempt, and suicidal consideration) were significantly associated with substance use.

**Table 2:** Background characteristics of the respondents by substance use.

		Substance use		
Variables	Total *n* (weighted %)	No *n* (weighted %)	Yes *n* (weighted %)	*p*-value
Age (years)				
<15	52,006 (54.4)	37,144 (59.1)	14,862 (37.0)	
15–17	64,242 (41.7)	33,768 (37.9)	30,474 (55.7)	
18+	6,383 (3.9)	3,385 (3.0)	2,998 (7.3)	<0.001
Sex				
Male	55,932 (48.0)	31,937 (43.6)	23,995 (63.8)	
Female	66,075 (52.0)	42,037 (56.4)	24,038 (36.2)	<0.001
Hunger				
No	88,556 (66.4)	52,284 (66.3)	36,272 (66.5)	
Yes	27,295 (33.6)	17,747 (33.7)	9,548 (33.5)	0.847
Number of close friends				
None	7,232 (3.9)	4,616 (3.6)	2,616 (5.0)	
One	12,335 (7.6)	7,897 (7.3)	4,438 (8.6)	
Two	18,232 (9.9)	10,598 (9.5)	7,634 (11.5)	
Three or more	83,840 (78.6)	50,744 (79.6)	33,096 (74.9)	<0.001
Physical activity				
No	29,437 (32.0)	20,142 (33.7)	9,295 (25.9)	
Yes	92,252 (68.0)	53,623 (66.3)	38,629 (74.1)	<0.001
Peer support				
No	31,071 (20.5)	17,420 (18.2)	13,651 (28.8)	
Yes	90,904 (79.5)	56,528 (81.8)	34,376 (71.2)	<0.001
Loneliness				
No	69,293 (54.6)	44,812 (56.1)	24,481 (49.1)	
Yes	53,429 (45.4)	29,596 (43.9)	23,833 (50.9)	<0.001
Worry				
No	74,246 (61.5)	48,705 (63.9)	25,541 (52.5)	
Yes	48,260 (38.5)	25,577 (36.1)	22,683 (47.5)	<0.001
Bullied				
No	72,097 (66.9)	45,905 (70.0)	26,192 (55.5)	
Yes	50,295 (33.1)	28,377 (30.0)	21,918 (44.5)	<0.001
Suicidal plan				
No	105,227 (90.8)	66,938 (92.8)	38,289 (83.5)	
Yes	17,257 (9.2)	7,420 (7.2)	9,837 (16.5)	<0.001
Suicidal attempt				
No	107,984 (91.9)	68,488 (94.3)	39,496 (83.1)	
Yes	14,542 (8.1)	5,836 (5.7)	8,706 (16.9)	<0.001
Suicidal consideration				
No	102,969 (90.4)	66,183 (92.7)	36,786 (82.0)	
Yes	19,052 (9.6)	7,992 (7.3)	11,060 (18.0)	<0.001

### Prevalence of substance use and injuries

**Figure 1:**
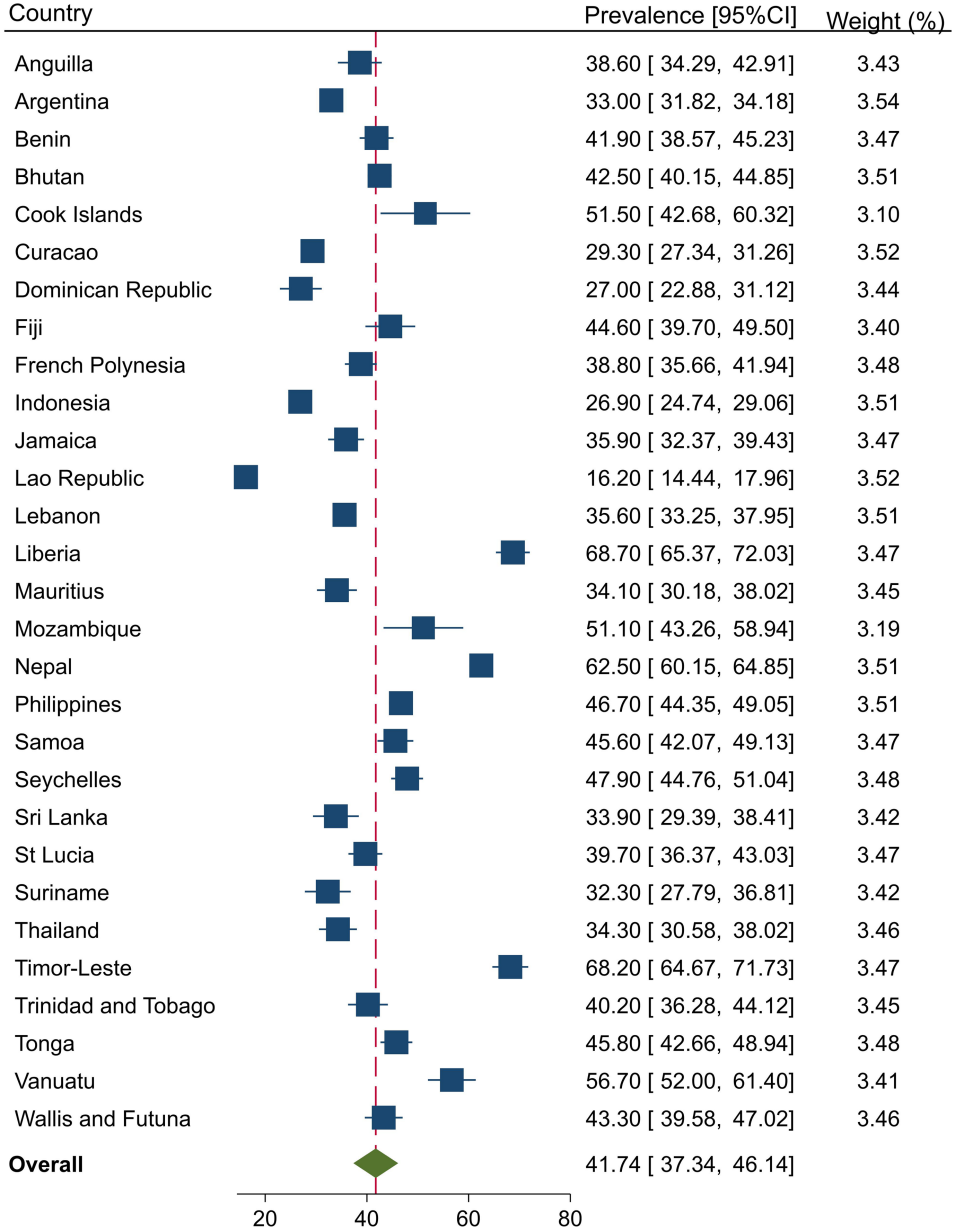
Country-specific and overall prevalence of injuries among in-school adolescents.

**Figure 2:**
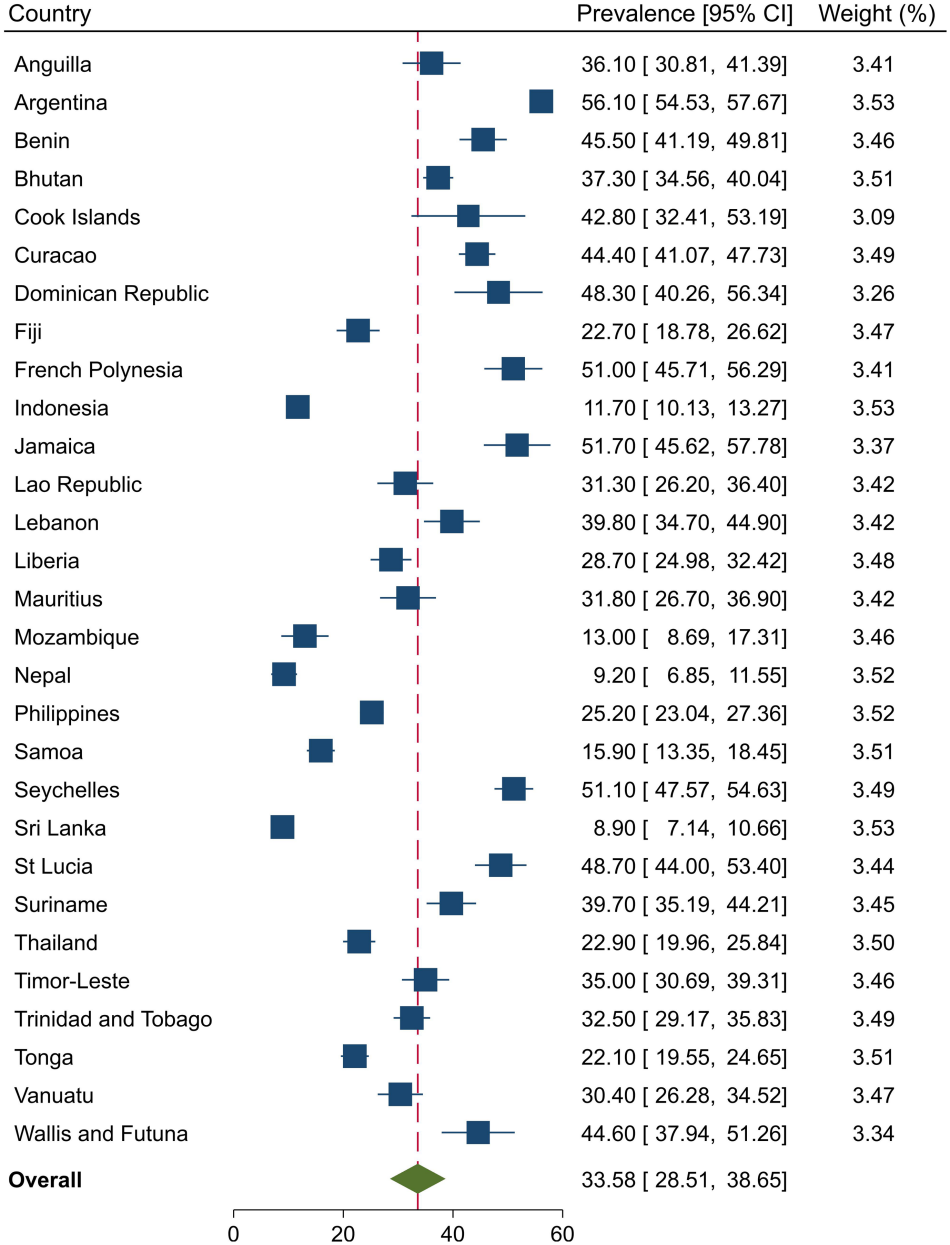
Country-specific and overall prevalence of substance use among in-school adolescents.

Figures 1, 2 present the country-specific prevalence of injuries and substance use, respectively. Overall, the injury prevalence was 41.7% (95%CI = 37.3, 46.1) among in-school adolescents and was significantly higher in males than females (47.3% vs. 36.4%, *p* = 0.001), respectively. The overall pooled prevalence of substance use was 33.6 (95%CI = 28.5, 38.6). Substance use was significantly higher among males than females (37.8% vs. 29.4%, *p* = 0.001), respectively.

### Influence of substance use on injuries and other risk factors associated with injuries

Table 3 shows the influence of substance use on injuries among in-school adolescents. It also presents other risk factors associated with injuries. When unadjusted, the odds of injuries were 94% higher among in-school adolescents who used substances than their counterparts who did not use substances [OR = 1.94, 95%CI = 1.80, 2.09]. When the effect of substance use on injuries was adjusted for all other variables, the odds of injuries were 40% higher among in-school adolescents who used substances [AOR = 1.40, 95%CI = 1.31, 1.49].

**Table 3:** Logistic regression analysis showing the association of substance use and risk factors with adolescents’ serious injuries.

Variable	OR (95%CI)	*p*-value	*AOR (95%CI)	*p*-value
Substance use				
No	1		1	
Yes	1.94 (1.80, 2.09)	0.001	1.40 (1.31, 1.49)	0.001
Parental supervision				
No	1		1	
Yes	0.81 (0.74, 0.88)	0.001	0.96 (0.89, 1.03)	0.266
Substance use x parental supervision				
Yes x Yes			1.14 (0.98, 1.32)	0.092
Age (years)				
<15	1		1	
15–17	1.06 (0.95, 1.17)	0.296	0.93 (0.86, 1.00)	0.053
18+	1.29 (1.12, 1.49)	0.001	0.92 (0.80, 1.05)	0.233
Sex				
Male	1		1	
Female	0.58 (0.55, 0.62)	0.001	0.58 (0.54, 0.62)	0.001
Hunger				
No	1		1	
Yes	1.56 (1.44, 168)	0.001	1.36 (1.26, 1.46)	0.001
Number of close friends				
None	1		1	
One	0.99 (0.85, 1.17)	0.965	1.17 (0.99, 1.38)	0.067
Two	0.94 (0.80, 1.11)	0.494	1.12 (0.95, 1.31)	0.180
Three or more	0.78 (0.66, 0.92)	0.004	1.03 (0.89, 1.20)	0.647
Physical activity				
No	1		1	
Yes	1.07 (1.01, 1.13)	0.032	1.03 (0.97, 1.09)	0.236
Peer support				
No	1		1	
Yes	0.67 (0.61, 0.74)	0.001	0.82 (0.75, 0.88)	0.001
Loneliness				
No	1		1	
Yes	1.60 (1.50, 1.71)	0.001	1.28 (1.20, 1.37)	0.001
Worry				
No	1		1	
Yes	1.71 (1.60, 1.82)	0.001	1.38 (1.29, 1.48)	0.001
Bullied				
No	1		1	
Yes	3.06 (2.80, 3.34)	0.001	2.54 (2.35, 2.74)	0.001
Suicidal plan				
No	1		1	
Yes	1.87 (1.70, 2.05)	0.001	0.96 (0.85, 1.09)	0.553
Suicidal attempt				
No	1		1	
Yes	2.83 (2.53, 3.17)	0.001	1.77 (1.58, 2.00)	0.001
Suicidal consideration				
No	1		1	
Yes	2.14 (1.95, 2.36)	0.001	1.25 (1.12, 1.40)	0.001

OR, Crude Odds ratio; AOR, Adjusted Odds ratio; CI, Confidence Interval. *Adjusted for substance use, parental supervision, age, sex, hunger, number of close friends, physical activity, peer support, loneliness, worry, bullying, suicidal plan, suicidal attempt, and suicidal consideration.

### Mediation effect of perpetration of and victimization by interpersonal violence on the association between substance use and injuries

**Figure 3:**
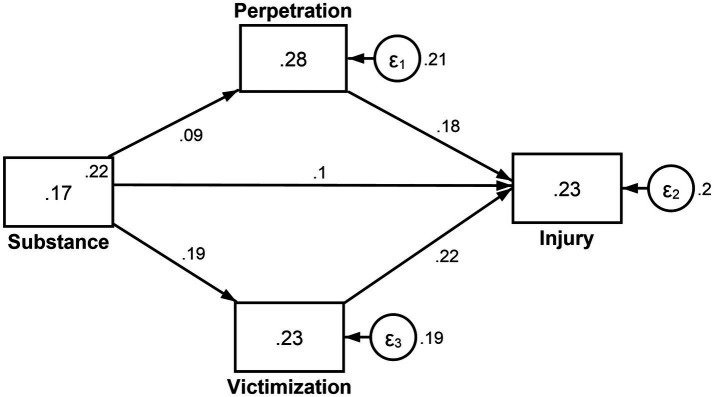
Mediation effect of perpetration of and victimization by interpersonal violence on the association between substance use and injuries.

Figure 3 presents a path analysis showing how perpetration of and victimization by interpersonal violence potentially mediates the effect of substance use on injuries. It revealed a positive direct effect of substance use on injuries [*β =* 0.10; 95%CI = 0.09, 0.11; *p* = 0.001]. Decomposition of the effects revealed a positive indirect effect of substance use on injuries running through perpetration of and victimization by interpersonal violence [*β =* 0.06; 95%CI = 0.05, 0.07; *p* = 0.001] with a total positive effect of both perpetration [*β =* 0.18; 95%CI = 0.16, 0.19; *p* = 0.001] and victimization on injuries [*β =* 0.22; 95%CI = 0.21, 0.24; *p* = 0.001]. This finding indicates a significant mediation effect of perpetration of and victimization by interpersonal violence on the association between substance use and injuries.

### Associations between substance types (tobacco, cigarette, alcohol, and marijuana) and forms of injuries

Table 4 presents a subgroup analysis showing the association between substance types and forms of injuries using multinomial logistic regression analysis. Compared to adolescents who do not use tobacco, those who used tobacco were 3.98 times more likely to sustain gunshot wound [OR = 3.98, 95%CI = 2.05, 7.75] rather than broken bone/dislocated joint. Also, compared to those who did not use cigarettes, adolescents who smoked cigarettes had 45% lower odds of sustaining a cut/stab wound rather than a broken bone/dislocated joint [OR = 0.55, 95%CI = 0.44, 0.68]. Moreover, compared to non-smokers of marijuana, adolescents who smoked marijuana had 2.81 higher odds of sustaining a gunshot wound [OR = 2.81, 95%CI = 1.29, 6.10] rather than broken bone/dislocated joint. Also, compared to non-users of alcohol, alcohol users were 25% less likely to sustain cut/stab wound [OR = 0.75, 95%CI = 0.62, 0.91], 53% more likely to sustain concussion/head injury [OR = 1.53, 95%CI = 1.19, 1.97], and two and half times more likely to sustain gunshot wound [OR = 2.50, 95%CI = 1.52, 4.12] rather than broken bone/dislocated joint.

**Table 4:** Multinomial logistic regression analysis of the associations between substance types (tobacco, cigarette, alcohol, and marijuana) and forms of injuries.

Injury types	OR (95%CI)	*p*-value
Broken bone/Dislocated joint	Base outcome	
Cut/Stab wound		
Tobacco use*	0.96 (0.73, 1.27)	0.797
Cigarette use*	0.55 (0.44, 0.68)	0.001
Marijuana use*	1.11 (0.84, 1.48)	0.456
Alcohol use*	0.75 (0.62, 0.91)	0.004
Concussion/Head injury		
Tobacco use*	1.43 (0.96, 2.13)	0.076
Cigarette use*	0.93 (0.70, 1.23)	0.617
Marijuana use*	1.57 (0.91, 2.70)	0.104
Alcohol use*	1.53 (1.19, 1.97)	0.001
Gunshot wound		
Tobacco use*	3.98 (2.05, 7.75)	0.001
Cigarette use*	1.57 (0.89, 2.77)	0.122
Marijuana use*	2.81 (1.29, 6.10)	0.009
Alcohol use*	2.50 (1.52, 4.12)	0.001
Bad burn		
Tobacco use*	0.75 (0.46, 1.23)	0.261
Cigarette use*	0.71 (0.49, 1.02)	0.064
Marijuana use*	0.63 (0.33, 1.19)	0.153
Alcohol use*	1.08 (0.81, 1.44)	0.582
Poisoned		
Tobacco use*	0.90 (0.46, 1.76)	0.768
Cigarette use*	0.88 (0.42, 1.84)	0.744
Marijuana use*	1.86 (0.85, 4.06)	0.121
Alcohol use*	1.56 (0.90, 2.72)	0.112

*Reference category is no. OR, Odds ratio; CI, Confidence interval.

## Discussion

To the best of our knowledge, this is the first study examining the mediation effect of both perpetration of and victimization by interpersonal violence on the association between substance use and severe injuries among adolescents from multiple countries. Even though the present study is distinct on this critical topic, previous studies have examined the direct relationships between substance use and serious injuries (26, 27). For example, Guvendeger Doksat et al. (26) reported a strong association between substance use, such as cannabis and cocaine, and non-suicidal self-injury. Van den Ban et al. have also revealed that severe injuries associated with hospital admissions among children and adolescents are twice as high in substance users (27). The current study extends the previously published literature by estimating the mediating role of perpetration of and victimization by interpersonal violence in the global context.

Although the exact mechanisms linking substance use to severe injuries are unclear, several plausible mechanisms and hypotheses have been proposed to account for the observed relationship between substance use and severe injuries among adolescents (10, 13). Our findings suggest that the involvement in interpersonal violence as a perpetrator and/or a victim mediates the connection between substance use and injury among in-school adolescents. Several reasons may explain why perpetration of and victimization by interpersonal violence could be a potential pathway linking substance use and severe injuries among adolescents. Existing evidence suggests that substance use may trigger violent and aggressive behaviours, such as physical attacks and fights which may lead to injuries (28, 29). Consequently, violent and aggressive adolescents may be entangled with other stressful and psychologically detached circumstances, potentially leading to severe injuries (30). The study further explains that correlates of substance use, such as low self-esteem, depression, and anxiety, could influence adolescents to engage in violent and aggressive behaviours, possibly leading to injuries (30). Thus, our findings support earlier evidence suggesting that substance use increases the likelihood of interpersonal violence, thereby intensifying the occurrence of injuries among adolescents. Dynamic strategic approaches involving all stakeholders, such as encouraging parental involvement in school activities, providing more creative, sports, and recreational activities centers for school-going adolescents, and improving on monitoring system in school to control substance use rates, are essential to tackle violent and aggressive behaviours in schools to address serious injuries further. The present study’s findings indicate that the double burden of substance use and interpersonal violence could negatively affect adolescents, which calls for attention both within and outside the school environment.

Lastly, the subgroup analysis yielded noteworthy results, indicating that certain substances, namely tobacco, marijuana, and cigarettes, exhibited statistically significant associations with specific types of injuries, including head injuries and gunshot wounds. For instance, our findings showed that tobacco users had a 3.98 times higher likelihood of sustaining a gunshot wound. Similarly, marijuana users had 2.81 times higher odds of sustaining gunshot wounds, and alcohol users had 1.53 and 2.50 times higher odds of sustaining head injury and gunshot wounds, respectively, underscoring the substantial impact of substance use on the severity of serious injuries. Previous studies have reported similar effects of marijuana and substance use on head injuries (31–33). These findings hold significant implications for public health, policy, and practice. They also emphasize the need for further clinical research to deepen our understanding of the relationship between substance use and injury occurrence, particularly among adolescents.

There are specific strengths and limitations to the present study. Our sample used nationally representative datasets with large sample sizes across several countries and territories, increasing the study findings’ generalizability. Despite these strengths, our results should be interpreted in the context of the following limitations. Although we adjusted for most known potential confounders, it is still possible that residual confounding may exist that impact or explain the results. Also, there is a potential timeframe imbalance between the measures. However, this may not be a methodological concern in a cross-sectional study design like the present study. Moreover, this cross-sectional study cannot infer causal relationships and implications from the association between substance use and serious injuries.

## Conclusion

This multi-context study among in-school paediatric populations has provided evidence showing a statistically significant association between substance use and serious injuries, which is mediated by perpetration of and victimization by interpersonal violence. Our results may have utility in informing future public health or substance use and interpersonal violence control policies and interventions, as well as strategies within and outside the school environment to address adolescent substance use and interpersonal violence.

## Data availability statement

Publicly available datasets were analyzed in this study. This data can be found here: http://www.cdc.gov/gshs/.

## Ethics statement

Ethical review and approval was not required for the study on human participants in accordance with the local legislation and institutional requirements. Written informed consent to participate in this study was provided by the participants’ legal guardian/next of kin.

## Author contributions

BA and PP had full access to all of the data in the study and took responsibility for the integrity of the data, the accuracy of the data analysis, acquisition, and analysis, or interpretation of data. PP: concept of design. BA: statistical analysis. BA, MA, SA-A, RO, SN, LM, AA, EO, CA, and PP: drafting of the manuscript and critical revision of the manuscript for important intellectual content. PP and CA: supervision. All authors contributed to the article and approved the submitted version.

## Conflict of interest

The authors declare that the research was conducted in the absence of any commercial or financial relationships that could be construed as a potential conflict of interest.

## Publisher’s note

All claims expressed in this article are solely those of the authors and do not necessarily represent those of their affiliated organizations, or those of the publisher, the editors and the reviewers. Any product that may be evaluated in this article, or claim that may be made by its manufacturer, is not guaranteed or endorsed by the publisher.

## Author disclaimer

The findings and conclusions of this report are those of the authors and do not necessarily represent the official position of the Centers for Disease Control and Prevention.
